# Nutrition and Healthy Ageing: Calorie Restriction or Polyphenol-Rich “MediterrAsian” Diet?

**DOI:** 10.1155/2013/707421

**Published:** 2013-08-28

**Authors:** Kathrin Pallauf, Katrin Giller, Patricia Huebbe, Gerald Rimbach

**Affiliations:** Institute of Human Nutrition and Food Science, Christian-Albrechts University of Kiel, Hermann-Rodewald-Straße 6, 24118 Kiel, Germany

## Abstract

Diet plays an important role in mammalian health and the prevention of chronic diseases such as cardiovascular disease (CVD). Incidence of CVD is low in many parts of Asia (e.g., Japan) and the Mediterranean area (e.g., Italy, Spain, Greece, and Turkey). The Asian and the Mediterranean diets are rich in fruit and vegetables, thereby providing high amounts of plant bioactives including polyphenols, glucosinolates, and antioxidant vitamins. Furthermore, oily fish which is rich in omega-3 fatty acids is an important part of the Asian (e.g., Japanese) and also of the Mediterranean diets. There are specific plant bioactives which predominantly occur in the Mediterranean (e.g., resveratrol from red wine, hydroxytyrosol, and oleuropein from olive oil) and in the Asian diets (e.g., isoflavones from soybean and epigallocatechin gallate from green tea). Interestingly, when compared to calorie restriction which has been repeatedly shown to increase healthspan, these polyphenols activate similar molecular targets such as Sirt1. We suggest that a so-called “MediterrAsian” diet combining sirtuin-activating foods (= sirtfoods) of the Asian as well as Mediterranean diet may be a promising dietary strategy in preventing chronic diseases, thereby ensuring health and healthy ageing. Future (human) studies are needed which take the concept suggested here of the MediterrAsian diet into account.

## 1. Introduction

It has been well established that diet plays a central role in maintaining health throughout life. In the Western world diet has significantly changed in recent years. Diets that are rich in saturated fats and cholesterol, low in fibre, and high in sugar and salt may increase the CVD risk [1–3]. We are currently facing an increase in the incidence of obesity associated diseases in general and specifically coronary heart disease in many parts of the world. Contrarily, a reduction of food and/or calorie intake without malnutrition may be associated with the prolongation of life- and healthspan. The National Academies Keck Futures Initiative; the Future of Human Healthspan, defined healthspan as the time an organism was of good health with health being the “the ability of a system to maintain or return to homeostasis in response to challenges” [4]. In mammals including primates, calorie restriction (CR) repeatedly delayed disease onset, thereby prolonging healthspan [5–7]. As diet composition also seems to influence health [8, 9], it has been hypothesized that CR and certain dietary factors can prevent age-related diseases like atherosclerosis and cardiovascular disease (CVD), type 2 diabetes mellitus, neurodegenerative diseases, and cancer. Dietary factors that decrease CVD risk may be the consumption of red wine and olive oil in the Mediterranean area [10] or seaweed in coastal Asia [11]. Interestingly, polyphenol consumption and calorie restriction (CR) seem to have similar effects on metabolism in humans [12, 13], and supplementation with polyphenols may attenuate negative effects from a high fat diet in mice [14]. Therefore, polyphenols are also referred to as CR mimetics. An important mechanism by which polyphenols induce CR-like signalling pathways seems to function through sirtuin activation [15]. Sirtuins are NAD-dependent deacetylases that were shown to be involved in the regulation of lifespan and metabolism in different organisms [15, 16]. Further evidence exists supporting a role of polyphenols in gene regulation via the gamma coactivator 1-alpha (PGC-1*α*) and transcription factors such as nuclear factor-erythroid 2 (Nrf2), forkhead box O (FoxO), and nuclear factor kappa B (NF*κ*B) [17], all of which are also implicated in CR-mediated effects. In this review we will discuss the literature on CR and foods that induce CR-like effects with a special focus on sirtuin activators that are found in the Asian and Mediterranean diets and their potential to promote healthy ageing. Because of their sirtuin-activating properties we refer to these foodstuffs as "sirtfoods".

## 2. Signalling Pathways That Are Induced by Calorie Restriction

CR induces body weight loss and has beneficial effects on plasma levels of triglycerides and cholesterol as well as on blood pressure, thereby preventing or delaying the onset of age-related diseases [18]. Additionally, in response to a reduction of body fat, adipose tissue-derived hormones including leptin and adiponectin are modulated. These hormones are central in the regulation of satiety and appetite in the hypothalamus [19]. As an important regulator of body weight [20], circulating leptin levels are directly proportional to the total amount of visceral adipose tissue. While CR decreases leptin levels, it increases adiponectin levels. In contrast to leptin, adiponectin exhibits antiatherogenic, anti-inflammatory, and insulin-sensitizing properties and is assumed to be cardioprotective [21]. 

### 2.1. Insulin-Like Growth Factor and Mammalian Target of Rapamycin Signalling

One of the main impacts of dietary restriction is the modulation of insulin and insulin-like growth factor-1 (IGF-1) signalling [22]. Interestingly, in mice impaired IGF-1 signalling leads to a similarly prolonged lifespan as compared to CR [23] and in *Caenorhabditis elegans* inactivation of the IGF/PI3K/Akt pathway promoted longevity [24].

Upon binding to their receptors, insulin and IGF-1 induce a number of signalling pathways and kinases such as the phosphatidylinositol 3-kinases class I (PI3KI), the serine/threonine kinase Akt, and the mammalian target of rapamycin (mTOR). mTOR promotes protein synthesis and cellular growth [25]. Besides downregulating mTOR via lowering IGF/PI3K/Akt signalling, CR also inhibits mTOR through activation of the 5′ adenosine monophosphate-activated protein kinase (AMPK) [26]. Inhibition of mTOR leads to prolongation of lifespan [27] which is in part mediated by autophagy, a lysosomal degradation process that degrades no-longer-needed proteins and organelles and also functions as a starvation response [28]. Consistent with CR attenuating age-related changes in metabolism and cellular functions, insulin sensitivity and autophagic activity decrease with age while being increased by CR [29]. 

### 2.2. Reactive Oxygen Species and the Hormesis Effect

Ageing is also characterized by accumulating oxidative damage. There are different mechanisms that lead to this build-up of damaged proteins, lipids, and organelles. Damaged macromolecules may accumulate because of reduced degradation, reduced antioxidant capacity, or increased reactive oxygen species production [30, 31].

It was shown that CR can decrease the mitochondrial release of reactive oxygen species (ROS) which contribute to this type of damage in ageing organisms [32]. However, recent studies have indicated that the metabolic rate increases with CR [33], and it has been hypothesized that deleterious substances including ROS may have beneficial effects at low concentrations. This concept of a biphasic dose-response with low and high doses of a substance mediating opposite effects is referred to as hormesis [34]. According to this concept, low doses of a certain substance benefit the organism by inducing adaptive effects. In line with the concept of hormesis, ROS may function as essential signalling molecules, for example, by regulating redox-sensitive transcription factors [35]. Because of a lack of energy supply under CR, mitochondrial activity and, as a consequence, oxidative phosphorylation in the respiratory chain could be enhanced, leading to increased ROS production [36].

### 2.3. The Redox-Regulated Transcription Factors Nrf2 and NF*κ*B

One of the targets of increased ROS production is the nuclear factor-erythroid 2- (NFE2-) related factor (Nrf2). This Cap'n'Collar basic leucine zipper transcription factor controls the expression of a large number of antioxidant and phase II detoxifying enzymes such as the NAD(P)H dehydrogenase (quinone) 1 (NQO1), the heme oxygenase 1 (HO-1), glutathione *S*-transferase (GST), the glutathione peroxidase (GPx), and the *γ*-glutamylcysteine synthetase (*γ*GCS) [37–39]. In CR rodents, upregulation and increased activity of these gene products have been described repeatedly [40, 41]. 

The nuclear factor kappa B (NF*κ*B) is also a redox-sensitive transcription factor that induces the expression of genes involved in inflammation and cellular proliferation [42]. Although NF*κ*B is activated by ROS, various studies have reported downregulation of NF*κ*B by CR [43, 44]. It has been suggested that CR increases cytoplasmic levels of I*κ*B which impedes NF*κ*B translocation into the nucleus. Additionally, CR may influence NF*κ*B translocation by decreasing nucleophosmin (NPM) expression. NPM is a nuclear phosphoprotein that shuttles between the nucleus and the cytoplasm and promotes NF*κ*B activity [45]. Moreover, CR may inhibit the transcription of the NF*κ*B subunit RelA/p65 through Sirt1 activation [46]. Considering that at a higher age NF*κ*B activity and as a consequence inflammation seem to increase [47], the down-regulation of NF*κ*B activity by CR may contribute to the ageing-related health benefits of CR.

### 2.4. Forkhead Box O Transcription Factors

The forkhead box O (FoxO) transcription factor family activates or represses gene expression of a wide variety of genes implicated in apoptosis, cell cycle and differentiation, DNA repair, and stress response [48, 49] and is also activated by CR [50]. Of the four human forkhead genes that have been identified so far (FoxO1, FoxO3, FoxO4 and FoxO6), FoxO3 has been identified as a longevity-associated gene in centenarians [51]. It has been suggested that FoxO transcription factors may be involved in longevity because of their ability to detoxify ROS and repair DNA damage. 

FoxOs can be regulated by various mechanisms such as phosphorylation, acetylation, and proteasomal degradation. While under nutrient rich conditions, insulin/IGF-1 signalling leads to translocation of FoxO out of the nucleus and subsequent degradation, and CR leads to activation of FoxO-mediated transcription [50]. An important mechanism for FoxO activation under conditions of limited nutrient supply is its deacetylation by Sirt1 [52] (see Table 1). It has been shown that Sirt1 leads to a type of FoxO3 activation that counteracts oxidative stress while suppressing FoxO3-induced apoptosis rather than leading to FoxO3-induced cell death [53]. The finding that Sirt1 can inhibit the transcription of one set of genes while switching on the transcription of another type of target genes could explain the observation made by Motta and colleagues [54] who stated that Sirt1-mediated deacetylation of FOXO3 led to its inhibition. However, in the case of FoxO1, Daitoku and coworkers [55] reported an upregulation via Sirt-mediated deacetylation. Interestingly, FoxO and the tumour suppressor p53 appear to be functionally linked as they can alter each other's functions [56]. Both FoxO and p53 are deacetylated by Sirt1, which thereby controls their activities. In addition, FoxO induces gene expression of Sirt1 [57]. Therefore, it seems plausible that CR, at least in part, leads to lifespan extension through regulating the insulin/IGF1, FoxO, and Sirt1 network.

## 3. Sirtuins, the Mammalian Homologues of the Yeast Longevity Gene SIR2

Sirtuins (Sirt1–7) are NAD-dependent histone deacetylases [59]. All sirtuins contain a catalytic core domain but differ in the protein sequences surrounding this domain and their cellular localization [60]. They act as protein deacetylases and/or ADP-ribosyltransferases. As they require NAD, it has been suggested that their activity depends on the metabolic state of the cell. It has been hypothesized that sirtuins may link energy intake to lifespan [61]. An inhibitory mutation of the Sirt1 ortholog SIR2 in yeast shortened lifespan, whereas overexpression of SIR2 extended lifespan [62]. Their cellular localizations, activities, and biological functions are listed in Table 2.

### 3.1. Sirt1 and the Control of Metabolism

**Figure 1:**
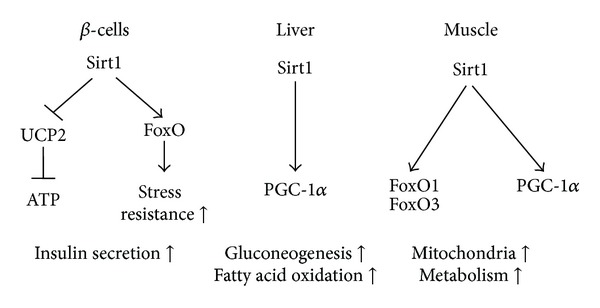
Sirt1-mediated regulation of metabolism in different tissues according to [92]. In pancreatic *β*-cells Sirt1 represses the expression of UCP2, thereby increasing insulin secretion, and Sirt 1 also regulates FoxOs, thereby protecting the *β*-cells against oxidative stress. In the liver Sirt1 regulates gluconeogenesis by activating PGC-1*α*. In muscle cells Sirt1 activates both PGC-1*α* and FoxO, thereby influencing mitochondrial biogenesis, respiration, and fatty acid oxidation. ATP: adenosine triphosphate; FoxO: forkhead box protein O; PGC-1*α*: peroxisome proliferator-activated receptor-*γ* coactivator 1 alpha; UCP: uncoupling protein 2.

Sirt1 is the best studied sirtuin to date and is sometimes referred to as a guardian against cellular oxidative stress and DNA damage. Apart from deacetylating p53 and FoxO transcription factors, it was also shown to interact with NF*κ*B, the peroxisome proliferator-activated receptor gamma (PPAR*γ*), and the PPAR*γ* coactivator and inducer of mitochondrial biogenesis PGC-1*α* [64, 65] (Figure 1). *In vivo* studies have shown that CR upregulates the mammalian Sirt1 protein levels in muscle, brain, fat, and kidney (see Table 3) [57, 66]. In white adipose tissue (WAT) Sirt1 was shown to deacetylate and inhibit PPAR*γ*. This nuclear receptor which is induced by fatty acids activates fat synthesis and adipogenesis. Thus, inhibition of PPAR*γ* by Sirt1 led to fat loss [64]. A decrease in adipose tissue generally lowers the leptin/adiponectin ratio, thereby favouring insulin sensitivity and healthy ageing. It has also been shown that Sirt1 activation shifts metabolisms away from using glucose as an energy source. While Sirt1 led to PPAR*γ* inhibition, PPAR*α* that transcribes genes involved in fatty acid oxidation was activated by Sirt1 localizing to PPAR response elements upon CR [67]. Additionally, deacetylation of PGC-1*α* by Sirt1 seems to lead to an activation of gluconeogenic gene transcription, while the expression of genes involved in glycolysis decreased [65]. With CR, PGC-1*α* is also induced on a transcriptional level and activated by AMPK-mediated phosphorylation [68]. Activated PGC-1*α* increases mitochondrial biogenesis by expressing several components of the respiratory chain. Moreover, PGC-1*α* contributes to the metabolic shift away from glycolysis under conditions of limited nutrient supply by promoting mitochondrial fatty acid oxidation and gluconeogenesis in the liver [69].

However, while various reports find increased Sirt1 protein levels in the muscle and WAT of CR animals, there is contradicting data as to whether liver Sirt1 changes upon CR (see Table 3). Chen and colleagues [66] even stated that hepatic Sirt1 levels were decreased by CR. This may be related to tissue-specific functions of Sirt1 (Figure 1). In the study by Chen and colleagues, the repression of hepatic Sirt1 by CR resulted in decreased hepatic fat synthesis and fat accumulation. Consistent with this finding, mice with a liver-specific knockout of Sirt1 fed a Western diet showed lower body weight gain than wild type mice. A possible explanation for this lowered fat accumulation in the liver may be a reduced coactivation of liver X receptor (LXR) resulting in decreased activation of the cholesterol transporter ABCA1 and the fat synthesis regulator SREBP1c [66]. However, in another study also using Sirt1 LKO contrary observations were made, and a weight gain in the LKO mice higher than in the wild type controls was reported [67]. As pointed out by Haigis and Sinclair [72], this may have resulted from the different types of diets used in these two studies, with the diet from the second trial containing more fat. Of interest, transgenic Sirt1 mice were protected against fatty liver when fed a high fat diet. Moreover, these mice showed lower NF*κ*B activation and, consistently, lower levels of proinflammatory cytokines as well as lower glucose levels when compared to their wild type controls [73]. Thus, the authors hypothesize that Sirt1 protects from damage induced by a high fat diet.

### 3.2. Cardiovascular Disease and Sirt1

CVD is the primary cause of death in most countries and is characterized by elevated levels of low density lipoprotein cholesterol, oxidative damage to proteins and lipids and chronic inflammation. This environment promotes atherosclerotic plaque formation and a deteriorated endothelial function. Apart from genetic factors, a high fat diet and ageing are the main risk factors for developing this type of disease. Sirt1 induction by CR or by the “sirtfood”- constituent resveratrol has been shown to counteract elevated levels of inflammation and might lower cholesterol and triglyceride synthesis [74]. An important factor that contributes to the protective role of Sirt1 in CVD seems to be its function as a suppressor of the proinflammatory transcription factor NF*κ*B. Sirt1 inhibits NF*κ*B-mediated transcription by deacetylating its subunit RelA/p65 and as a consequence lowering its DNA-binding ability [46]. Various reports have shown that Sirt1 activation *in vitro* decreased the levels of proinflammatory mediators such as the tumour necrosis factor *α*, interleukins 1 and 6, intercellular adhesion molecule 1, and inducible nitric oxide synthase (NOS) [72]. In a mouse model for CVD, an apolipoprotein E double knockout, and endothelial Sirt1 overexpression lowered the formation of atherosclerotic plaques [75]. In mouse liver, it could be shown that Sirt1 interacts with the nuclear factor LXR and activates this transcription factor through its deacetylation. LXR induces reverse cholesterol transport by activating the transcription of the cholesterol efflux transporter ABCA1, thereby lowering peripheral cholesterol levels and promoting cholesterol excretion [76]. In contrast to inducible NOS which is found in macrophages and produces NO that harms microorganisms, endothelial NOS (eNOS) protects arteries. Interestingly, it was shown that Sirt1 activates eNOS and thus leads to endothelium-dependent vasodilatation.

### 3.3. Sirt1 Interaction with AMPK and Importance for Autophagy

Activation of the energy sensing kinase AMPK has shown similar effects on metabolism energy expenditure as compared to sirtuin signalling on various occasions [77]. This can be explained by the findings that Sirt1 activates AMPK [78] and vice versa [79]. Additionally, these reports point to the notion that both enzymes are needed to orchestrate the organism's response to limited nutrient supply or elevated energy demand during exercise. A part of the organism's response to such catabolic metabolic states is the lysosomal degradation pathway autophagy. Interestingly, autophagy was shown to be induced via Sirt1 and AMPK [80, 81]. In *Caenorhabditis elegans*, autophagy induction is necessary for and contributes to the lifespan prolonging effect of CR and its mimetic resveratrol [82]. In another experiment measuring lifespan extension, autophagy deficient worms did not benefit from caloric restriction to the same extent as wild type *C. elegans* [83]. Similarly to the activation of sirtuins, autophagy induction leads to lifespan extension in *Drosophila* and other organisms [84, 85]. In addition, sirtuins and autophagy are induced by CR [29]. Therefore, part of the ageing-related benefits from Sirt1 activation by CR or polyphenolic CR mimetics seems to be caused by autophagy [86]. In the case of the secondary plant metabolite resveratrol this connection could already be shown. By inducing autophagy via Sirt1 activation, resveratrol promoted longevity in worms and flies [87]. Interestingly, other polyphenolic Sirt1 inducers such as quercetin also promoted autophagic degradation [15, 88]. Considering that autophagy is a starvation and especially in higher eukaryotes a stress response, the activation of this lysosomal degradation pathway might be one the main mechanisms by which Sirt1 protects the organism from stressors (and thereby contributes to longevity).

### 3.4. The Mammalian Sirtuins 2–6

Apart from Sirt1, both Sirt3 and Sirt4 play important metabolic roles during food limitation. Additionally, in a study by Kawahara and coworkers [89], Sirt6-induced histone deacetylation led to prolongation of lifespan.

**Figure 2:**
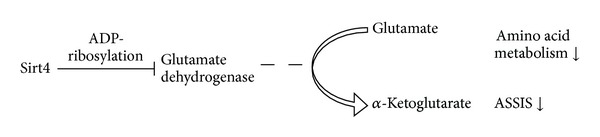
Sirt4 mediated amino acid metabolism according to [92]. ASSIS: amino acid-stimulated insulin secretion.

Sirt3 is mainly expressed in the liver, kidney, brain, and brown adipose tissue [90]. A high expression of Sirt3 in brown adipose tissue during CR induces the expression of PGC-1*α* and the uncoupling protein-1 (UCP-1) which is important for thermogenesis. Downregulation of Sirt4 by CR seems to affect amino acid metabolism and insulin secretion. Decreased Sirt4 levels lead to a higher conversion of glutamate to *α*-ketoglutarate by the glutamate dehydrogenase GDH [91], thereby promoting the use of amino acids as ATP source under CR. Contrarily, under nutrient-rich conditions Sirt4 ADP-ribosylates GDH which attenuates GDH activity [91]. Furthermore, CR-mediated Sirt4 decrease and GDH activity induce the amino acid-stimulated insulin secretion (AASIS) resulting in increased insulin secretion in response to CR [92] (Figure 2).

## 4. Potential Health Benefits of a MediterrAsian Diet

As in the Western world the prevalence of obesity and age-associated diseases such as type 2 diabetes mellitus, cancer, Alzheimer's, and atherosclerosis is increasing, and in order to benefit from CR a lifelong restriction seems to be necessary [41], interest in dietary restriction mimetics has been rising (see Table 4). 

However, although substances such as metformin can be used as an antidiabetic drug [97], most of the pharmacological CR mimetics have severe side effects. Therefore, the consumption of secondary plant bioactives such as polyphenols with the normal diet appears to be a safer strategy to benefit from potentially healthspan-improving CR mimetics. 

Although further studies are needed to prove that these foods promote health in humans through their CR-mimicking secondary plant metabolite content, we hypothesize that the concept of combining foods from Asian and Mediterranean diets could possibly improve health status in an ageing population. Unfortunately, it can be difficult to study how dietary components influence health because of the low doses of bioactives found in diets when compared to the doses fed in some animal studies or the doses applied in most cell culture models. Additionally, bioactives are metabolised before and after adsorption from the gastrointestinal tract and could work synergistically or antagonistically with each other and other components in the diet. These factors make it complicated to extrapolate findings from *in vitro* and *in vivo *experiments to humans. Furthermore, when carrying out human studies, dietary effects tend to be smaller than in many pharmacological intervention studies and therefore require a higher number of participants to obtain measurable results. However, it seems that diets rich in saturated fats and cholesterol, low in fibre, and high in sugar and salt may increase CVD risk [1–3], and dietary factors such as olive oil consumption in the Mediterranean area [10] or seaweed in coastal Asia [11] may decrease CVD risk. Therefore, we believe that combining Asian and Mediterranean foods in a MediterrAsian diet could be a promising approach for improving human health.

### 4.1. Components of Mediterranean Diets

**Figure 3:**
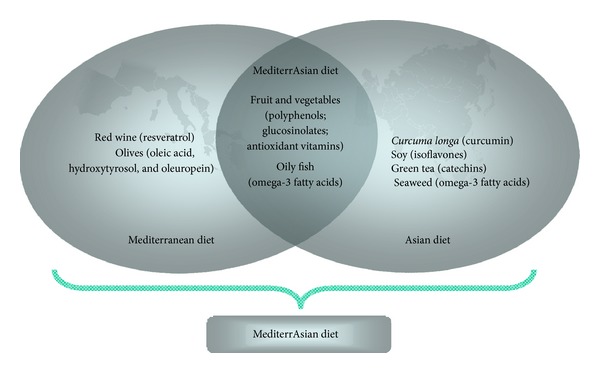
Important food items and their corresponding constituents in the Mediterranean, Asian, and so-called MediterrAsian diet.

From a nutritional point of view there is a substantial overlap between the Asian and Mediterranean diets. These diets are rich in fruit and especially vegetables which are both important sources of dietary polyphenols, glucosinolates, and vitamin C (see Figure 3). Furthermore, for some parts of Asia and the Mediterranean a high consumption of oily fish rich in omega 3 fatty acids has been reported. Consistent with the low incidence of CVD in populations consuming such diets in Asia and the Mediterranean area, it has been suggested that foods with a high content of polyphenols, glucosinolates, and omega 3 fatty acids reduce the CVD risk.

In addition, there are also specific foods which are predominantly consumed in the Mediterranean area such as red wine. The same is true for olive oil which is also consumed to a higher extent in Southern as opposed to Northern Europe. Studies in model organisms (e.g., *C. elegans*, *D. melanogaster*), and laboratory rodents suggest that red wine constituents (e.g., resveratrol) may positively affect both health and lifespan [14, 16]. Our own data demonstrate that mice consuming diets rich in olive oil phenolics (e.g., hydroxytyrosol) exhibit decreased oxidative damage markers (e.g., lipid peroxides, protein carbonyls) and improved expression of Nrf2-dependent genes encoding antioxidant (*γ*GCS, NQO1) and cardioprotective (paraoxonase) proteins. We have also shown that olive oil phenolics may induce proteasomal activity and Sirt1 signalling [98]. Recently, another group also observed Sirt1 induction by olive oil phenolics [99], and further evidence exists supporting a role of polyphenols in gene regulation via sirtuins and transcription factors such as Nrf2 and NF*κ*B [17]. Resveratrol activated Nrf2 and attenuated oxidative stress, thereby protecting the endothelium in a mouse model [100] and inhibited inflammation in macrophages via downregulation of the proinflammatory NF*κ*B [101]. Resveratrol has been repeatedly shown to induce Sirt1 [15, 82, 102], and therefore it seems possible that the endothelium protection and NF*κ*B inhibition may also be connected to Sirt1 induction. The polyphenol quercetin found in onions could also induce Sirt1 [15]. In Table 5, we have listed polyphenols that were shown to induce sirtuins and the type of foods they are found in. Given the fact that Sirt1 activation appears to benefit health by mimicking CR, these “sirtfoods” may contribute to healthy ageing.

### 4.2. Components of Asian Diets

The Asian diet is rich in soy and turmeric. *Curcuma longa* is a significant source of curcumin [111], and soy contains considerable amounts of isoflavones [112, 113]. Similar to resveratrol and quercetin, curcumin has also been shown to induce Nrf2 [114] and other transcription factors [115] and inhibit NF*κ*B-mediated inflammation [116, 117]. In contrast to the Asian diet, the Mediterranean diet is almost devoid of both isoflavones and curcumin. We and others have shown that soy isoflavones and curcumin mediates cardioprotective activity including reduction of LDL oxidation, inhibition of platelet aggregation, and improvement in vascular reactivity [118–120]. Interestingly, isoflavones such as daidzein were also shown to induce Sirt1 and PGC-1*α* [103]. Further constituents of the Asian diet that contain possible health-promoting bioactives are green tea and seaweed. Tea polyphenols seem to inhibit the proinflammatory transcription factor NF*κ*B [121], while seaweed is a source of antioxidant vitamins and polyunsaturated omega 3 fatty acids [122] which may also prevent CVD [123, 124]. 

### 4.3. Human Intervention Studies on Secondary Plant Metabolites Found in the MediterrAsian Diet

Most of the *in vitro* and* in vivo* research and various human intervention studies with bioactives from the Mediterranean and Asian diets found promising results regarding possible health-promoting benefits [125, 126]. However, some studies reported controversial data. For example, red wine has been stated to decrease low density lipoprotein (LDL) peroxidation in humans which would be beneficial for CVD prevention [127]. In contrast, red or white wine with a reduced alcohol content did not decrease (LDL) peroxidation in human volunteers [128], and one group of researchers showed that although moderate red wine consumption could lower LDL oxidation, the other tested alcoholic beverages also did [129]. On the other hand, in one study only red wine as compared to white wine lowered LDL oxidation in healthy humans, thereby pointing to the notion that certain components found in red but not white wine are responsible for this effect [130]. Similarly conflicting results were reported for the influence of fruit-derived polyphenols on blood pressure. While Aviram and colleagues observed that pomegranate juice reduced blood pressure in humans [131], in the study by Sumner and colleagues blood pressure did not change after administering pomegranate juice to the patients [132]. In the context of cancer prevention by soy-containing diets, a meta-analysis on the influence of isoflavones on a breast cancer biomarker found only a small positive effect that might or might not be clinically relevant [133]. In the case of curcumin, data from cell models and animal studies made this polyphenol appear an efficient treatment for dementia [134]. However, in clinical trials curcumin did not improve the symptoms of Alzheimer patients [135]. There are various factors that may contribute or explain these controversial findings including the differences in the health status of the patients, different doses and dosage forms, or different parameters measured (e.g., lipid peroxidation in plasma versus blood pressure for CVD). However, these conflicting results demonstrate that further research is needed to better understand the effect of dietary components on sirtuin activation and healthy ageing. 

## 5. Conclusion

A high content of fruit, vegetables, and oily fish in the diets of Mediterranean and certain Asian populations is likely to cause the improved health status observed in the MediterrAsian area. 

However, we suggest that plant bioactives, antioxidant vitamins, and omega-3 fatty acids do not work in isolation. Rather they may act synergistically, thereby preventing chronic diseases. Thus, it is possible that the complex mixture of diet-derived plant bioactives, antioxidatives, and omega-3 fatty acids cannot be substituted by a single purified compound [136]. 

Instead we propose combining healthy foodstuffs of the Asian and the Mediterranean diets especially rich in “sirtfoods” in order to prevent chronic diseases and ensure healthy ageing. 

We would like to encourage future studies in cultured cells, model organisms, laboratory rodents, and ultimately humans to unravel and evaluate potential health benefits of the MediterrAsian diet from a molecular to the system biology level.

## Figures and Tables

**Table 1:** FoxO regulation by Sirt1.

Cell culture	Treatment	Outcome	Reference
HEK 293T	Stress conditions (H_2_O_2_)	↑ Interaction between Sirt1 and FoxO3	[53]
MEF Sirt1^−/−^	Treatment with LY 294002 (PI3K inhibitor): wild type Sirt1^−/−^	↑ Acetylation of FoxO3 ↑ GADD45 (stress resistance) ↓ GADD45	
HepG2	Serum starvation => FoxO translocation into the nucleus	↑ Deacetylation of FoxO1 by Sirt1	[55]
HeLa	Sirt1 overexpression	↓ FoxO3 activity (↓ FoxO3 target genes Bim/p27)	[54]
HEK 293T	Inhibition of Sirt1	↓ FoxO4 activity ↓ MnSOD ↓ p27	[58]

Bim: Bcl-2 interacting mediator of cell death, proapoptotic protein; H_2_O_2_: hydrogen peroxide; HEK-293T: human embryonic kidney 293 cells containing the T antigen from simian virus; HepG2: human liver carcinoma cell line; MEF: mouse embryonic fibroblasts; MnSOD: manganese superoxide dismutase, part of antioxidative defence; p27: cyclin-dependent kinase inhibitor, controls cell cycle progression; PI3K: phosphatidylinositol 3-kinase.

**Table 2:** Cellular localization, activity, and biological function of sirtuins 1–7 according to [63].

Sirtuin	Localization	Activity	Biological function
Sirt1	Nucleus/cytosol	Deacetylase	Cell survival/metabolism
Sirt2	Cytosol	Deacetylase	Cell cycle
Sirt3	Mitochondria	Deacetylase	Thermogenesis/metabolism
Sirt4	Mitochondria	ADP-ribosyltransferase	Insulin secretion/metabolism
Sirt5	Mitochondria	Deacetylase	Unknown
Sirt6	Nucleus	ADP-ribosyltransferase	DNA repair
Sirt7	Nucleolus	Unknown	rDNA transcription

ADP: adenosine diphosphate; rDNA: ribosomal desoxyribonucleic acid; Sirt: sirtuin.

**Table 3:** Effects of calorie restriction or starvation on Sirt1 in different tissues in mice and rats.

Species	Number animals	Caloric restriction	Tissue	Outcome	Reference
Mice (C57BL6)	10	40%	Muscle, fat liver	Sirt1 ↑ (protein) Sirt1 ↓ (protein)	[66]
Mice (C57BL/6×C3H/He F1 hybrid)	4	15%	Brain, liver heart, muscle	No effect on Sirt1 (protein) Sirt1 ↓ (protein)	[13]
Mice (C57BL6)	—	24 h starvation	Liver	Sirt1 ↑ (protein) no effect on mRNA	[65]
Mice (C57BL)	—	24 h starvation	Brain, heart, muscle, white adipose tissue, and kidney	Sirt1 ↑ (protein) no effect on mRNA	[70]
Mice (C57BLK/6×SV127 F1 hybrid)	10	30–40%	White adipose tissue	Sirt1 ↑ (protein)	[36]
Mice (C57BL6)	—	40%	White adipose tissue, liver, kidney, and brain	Sirt1 ↑ (protein) not significant	[71]
Rats (Fisher 344)	—	40%	Brain, fat, kidney, and liver	Sirt1 ↑ (protein)	[57]

**Table 4:** Dietary restriction mimetics: mechanisms and side effects.

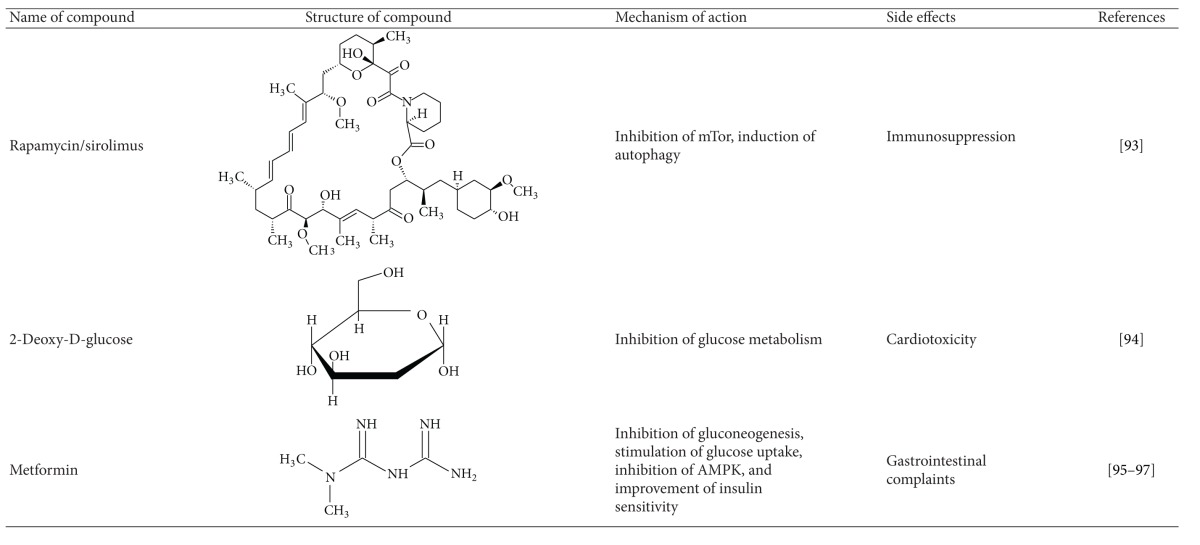

**Table 5:** Sirt1-inducing plant bioactives and the “sirtfoods” they are found in.

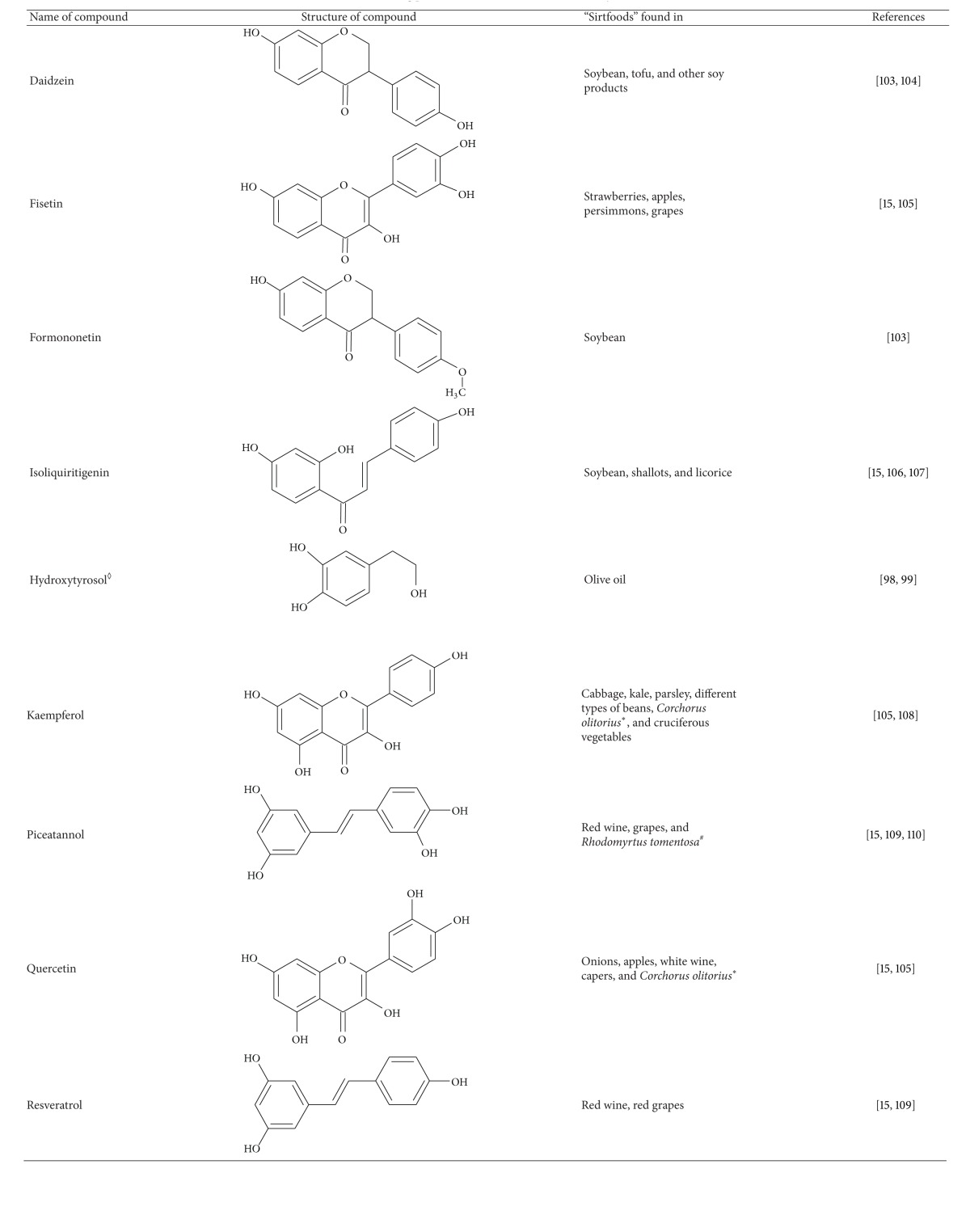

*consumed in Japan as “Molokheka”; ^#^edible plant native to Asia; ^◊^and possibly other phenolic compounds found in olive oil.
